# A Comparative Evaluation of Enamel Surface Roughness of Two Different Bonding Adhesives After Debonding With Atomic Force Microscopy

**DOI:** 10.7759/cureus.31661

**Published:** 2022-11-18

**Authors:** Swaroop Doddavarapu, Brahmani K, Gowri Sankar Singaraju, JS Yamini Priyanka, Ganugapanta Vivek Reddy, Prasad Mandava

**Affiliations:** 1 Orthodontics and Dentofacial Orthopaedics, Narayana Dental College, Nellore, IND; 2 Orthodontics and Dentofacial Orthopaedics, Vydehi Institute of Dental Sciences, Bangalore, IND

**Keywords:** tungsten, surface roughness, orthodontic, light cure, gic, enamel, debonding, composite, brackets, adhesive

## Abstract

Introduction

The debonding procedures should restore the enamel surface to its pre-treatment state as much as possible after removing orthodontic attachments and all remaining adhesive remnants from the surface of the tooth. The orthodontic attachments are bonded commonly by a light-cured composite system. Light-cured glass ionomer cement has been developed as an alternative to the composite as a bonding agent for orthodontic brackets. There are different methods for cleaning the residual adhesive after the removal of orthodontic attachments. The study aims to evaluate and compare the enamel surface roughness of teeth between two different adhesive systems - light cure composite and glass ionomer cement adhesives - after debonding followed by the removal of resin remnants with a tungsten carbide (TC) bur. A null hypothesis is proposed that there exists no difference in the enamel surface roughness between the two adhesive systems.

Materials and methods

The test sample of 40 freshly extracted human premolar teeth (n = 40) for orthodontic purposes was taken up for this in vitro study based on the inclusion and exclusion criteria. The sample was randomly assigned into two equal groups, with metal brackets bonded in Group-I (n = 20) by light cure adhesive (Trans bond XT, 3M Unitek, Monrovia, CA) and Group-II (n = 20) with light glass ionomer cement (GC Fuji Ortho LC, Tokyo, Japan). All samples were stored in water at room temperature for 24 h and brackets were debonded with a debonding plier. The removal of cement adhesive remnants was performed with a TC bur with a low-speed handpiece. The three surface roughness parameters, average roughness (Ra), root mean square roughness (Rq), and maximum roughness depth (Rmax), were measured at T1 (before bonding) and at T2 (after debonding and finishing) and the values were compared. The mode of bond failure was assessed by a modified adhesive remnant index (ARI) and the time required for the clean-up of adhesive was also noted.

Statistical analysis

The continuous quantitative data were statistically analyzed using SPSS Statistics v. 25.0 (IBM Corp., Armonk, NY). Student’s independent t-test/independent-samples t-test is an inferential statistical test for analyzing the difference between the two groups. Paired t-test was used for comparison within the group. The ARI between the groups was analyzed by a chi-square test. The probability (p) value for statistical significance was 0.05 or less for the difference between any two groups for all the analytical tests.

Results

A comparison of enamel surface roughness before bonding and after debonding for both groups revealed that there was a statistically significant difference within each group. The surface roughness values of composite resin - Ra (98.75 ± 0.96), Rq (120.38 ± 1.06), Rmax (650.14 ± 1.12) - and glass ionomer cement group - Ra (98.75 ± 0.96), Rq (62.76 ± 1.32), Rmax (434.36 ± 1.60) - show that there was a statistically significant difference between the groups with p <0.01.

Conclusion

There was a significant increase in the surface roughness of enamel after debonding of brackets and finishing with a TC bur with both the light cure and the glass ionomer cement adhesive systems. The light cure group showed more enamel surface roughness when compared to the glass ionomer cement group. In this study, the null hypothesis was rejected as there is a significant difference between the groups tested.

## Introduction

The direct bonding technique has revolutionized the field of orthodontics as a viable and excellent alternative to banding, and its popularity increased significantly over the years. The direct bonding procedures of brackets and other attachments should provide sufficient bonding strength to resist the orthodontic forces as well as the masticatory forces to prevent their failure during the treatment. On the other hand, during debonding of the brackets, this adhesion should cause minimal or no damage to the enamel. Achieving a smooth enamel surface after orthodontic bracket debonding is peremptory to prevent further untoward events such as plaque accumulation, discoloration, and other esthetic problems [1-7].

During bracket removal, bond failure can occur at different interfaces, adhesive failure at adhesive-enamel or adhesive-bracket interface, or due to cohesive failure within adhesive, depending upon the adhesive system and the procedures used for bonding of brackets. These procedures leave some adhesive on the enamel surface that should be removed by the orthodontist through finishing and polishing procedures [2-4].

It is imperative and obligatory for an orthodontist to choose a protocol that causes iatrogenic damage to the enamel. The variables that should be considered during these procedures include the adhesive system used for bonding, the type of curing of adhesive, debonding instruments, primary instruments, and materials for adhesive removal and enamel polishing agents.

Restoration of the enamel after orthodontic treatment includes two major steps: debonding and enamel surface polishing. Previous studies showed that there is a definite increase in enamel surface roughness [1-5] as well as an association with its discoloration affecting the esthetics [6,7]. The procedures and protocols involved after debonding should ideally restore the pretreatment condition of the enamel [2,3].

To our knowledge, most of the previous studies focused on the composite resin-based etch-and-rinse adhesive system [1-8]. Only a few studies [6-9] described the removal of resin-modified glass ionomer cement (RMGIC) after debonding. The other thing is that the subjective evaluations of scanning electron microscopy (SEM) images were performed without objective quantitative evaluation of the surface characteristics of the enamel resulting from these procedures. Only a few studies [10-12] evaluated the surface parameters of enamel by the three-dimensional objective method by atomic force microscope (AFM). 

Numerous finishing and polishing procedures were tested for their effect on the surface finish of the enamel. Finishing with diamond finishing bur or tungsten carbide (TC) bur remains the primary step in the polishing method and is a highly efficient surface cleaning method (least time-consuming). A recent systematic review established that the TC burs were faster and more effective than Sof-Lex discs, ultrasonic tools, hand instruments, rubbers, or composite burs but were less destructive than Arkansas stones, green stones, diamond burs, steel burs, and lasers [13].

In this study, we sought to compare the enamel surface roughness values by AFM method after orthodontic bracket debonding and adhesive removal using a TC bur when bonded with two different types of adhesive systems. A hypothesis was put forward that there exists no difference in the surface properties of enamel between the two adhesive systems after finishing with tungsten bur.

## Materials and methods

The protocol for this in vitro study was approved by Institutional Ethical Committee at Narayana Dental College, Nellore (Regd. No. D168408009; Ref No. NDC/IECC/ORT/10-16/53 dated 25/05/2016) and the experiment was conducted at the Indian Institute of Technology, Chennai, India. 

Test sample

The test sample consists of 40 freshly extracted healthy human premolar teeth for orthodontic treatment purposes. The teeth with periodontal lesions, carious lesions, hypoplastic defects, fractured or cracked teeth, or other morphological abnormalities were excluded.

Preparation of acrylic blocks

Initially, the extracted teeth were stored in distilled water, and the roots of the teeth were removed by using metallic disc bur, and the crowns were embedded in self-cured acrylic, with labial surfaces facing upwards. The teeth were cleaned and polished with non-fluoridated pumice, rinsed with water, and dried with oil-free compressed air.

Randomization and grouping

**Figure 1:**
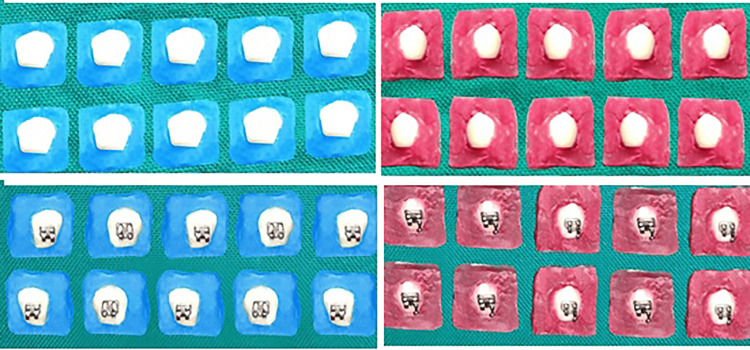
Color coding of acrylic blocks. Teeth bonded with metal brackets with light cure composite - blue (Group-I). Teeth bonded with metal brackets with RMGIC - red (Group-II). A representative first-batch sample (n = 10) for each group before and after bonding is shown. RMGIC: resin-modified glass ionomer cement.

The 40 selected teeth were randomly assigned by lot method to each of the two groups so that allocation followed equal sample distribution for the groups tested. The acrylic base in each of the test groups was color-coded (Figure 1). Group-I (n=20): teeth bonded with metal brackets with light cure composite were coded blue; and Group-II (n=20): teeth bonded with metal brackets with RMGIC were coded pink. The collection of samples and procedures were carried out in batches. The blocks were color-coded to prevent mixing up when sent for laboratory testing.

The initial measurement of surface roughness (T1)

**Figure 2:**
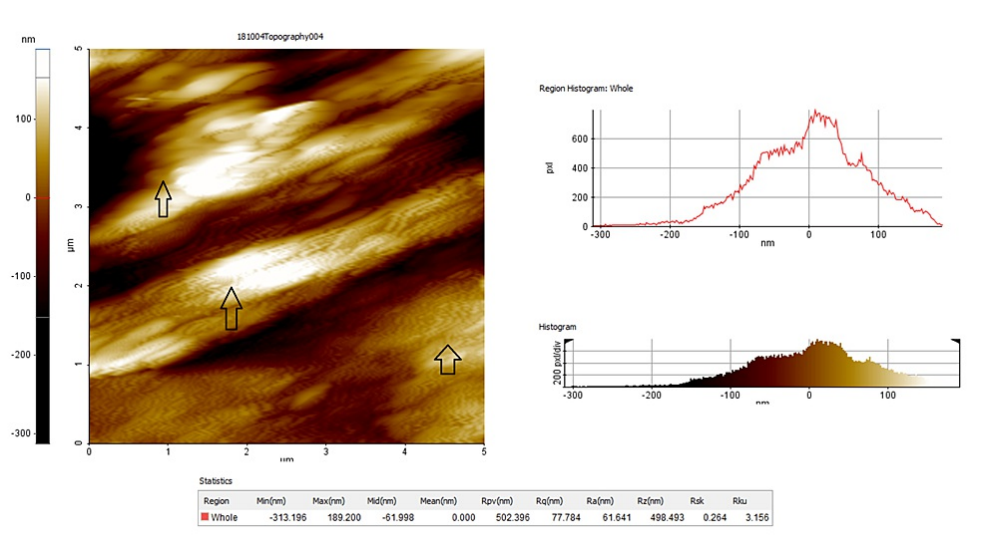
Topographic image and measurement of the enamel surface roughness characteristics before bonding (T1). Arrow marks showing the smooth surface of the enamel before bonding procedures

Before the initiation of bonding procedures, the three surface roughness parameters (average roughness (Ra), root mean square roughness (Rq), and maximum roughness depth (Rmax)) were evaluated by AFM (Digital Instruments MMAFM-2/1700EXL, Santa Barbara, CA), which was operated in the contact mode first to obtain topographic images over selected areas on the surface. This instrument supported a scanner with a maximum range of 100 µm x 100 µm x 5 µm in x, y, and z directions, respectively. Images were acquired with a scan rate of 2.03 Hz and 5 µm scan sizes. To measure roughness values, the tip was moved across the surface, and three different points were measured on the same surface located at the center of the sample (Figure 2).

Bonding procedures for Group-I

All the procedures including bonding, debonding, and clean-up were carried out by the primary Investigator (SD) under the direct guidance of a senior researcher (SGS). No blinding was done and the primary researcher is aware of the procedures.

The buccal surfaces of each crown (n=20) were cleaned with fluoride-free pumice and dried for 10 s. The labial surface of the crown was etched for 20 s with a 37% phosphoric acid gel (Restorite etching gel containing silica and 37% phosphoric acid, Prime Dental Products Pvt Ltd, Thane, India) and was thoroughly rinsed with water, and the tooth surface is dried with oil and moisture-free compressed air. A thin coat of primer (Orthosolo, universal bond enhancer, ORMCO Corporation, India) was applied, followed by light curing for 20 s (Mini S curing light, Guilin Woodpecker Instruments Pvt Ltd, Guilin, China). Stainless steel bracket (straight wire, MBT prescription - 0.22 slot) was applied with composite adhesive (Enlight light cure adhesive, Ormco Corporation, Orange, CA) and placed at the facial axis point (FA) on the mid-buccal surface of the tooth along the facial axis of the clinical crown (FACC) using the bracket-holding instrument. The excess composite around the bracket was removed. This was followed by light curing for 40 s from all the surfaces. All the samples were stored in water at room temperature for 24 h.

Bonding procedures for Group-II

After prophylaxis, the tooth was thoroughly rinsed with water and air-dried. There was no acid etching step in this group. The buccal enamel surface was conditioned with 10% polyacrylic acid for 20 s. The RMGIC bonding cement (GC Fuji Ortho Lc, Japan) is manipulated according to the manufacturer's instructions. Stainless steel bracket (straight wire, MBT prescription - 0.22 slot) was applied with a slightly thicker mix. The RMGIC adhesive was light-cured for a total of 40 s from all the surfaces. The bracket was placed in the same position on the labial surface of the tooth as described for Group-I. All the samples were stored in water at room temperature for 24 h.

Debonding process and evaluation of adhesive remnant index

All the brackets in both groups were debonded using a conventional debonding plier (GDC Bracket Remover # Straight Plier-3000/83). Debonding is done by the "wings model" as described by Brosh [14]. The tips of the pliers were placed under both sets of the occlusal and gingival wings and above the bracket base. The handles of the pliers were squeezed until the angled ends of the handles met, and the instrument was rotated toward the occlusal edge of the bracket until the bracket was removed.

The next step was the observation of debonded tooth surface under 60× magnification by using a scanning electron microscope ZEISS (Carl ZEISS, model EVO MA). A modified adhesive remnant index (ARI) as described by Bishara [15] was used to quantify the amount of adhesive on the tooth surface after debonding. Scoring was done based on the amount of resin material remnant on the tooth surface that is represented by counting the number of replicated mesh gauges present in the microscopic picture of debonded enamel.

Clean-up procedures

In both groups, the removal of adhesive remnants was performed with a 12-fluted carbide adhesive removal bur (prima classic-FG #FG-H379-023AGK) on a low-speed handpiece. The removal of the adhesive remnants was verified clinically by visual inspection under a dental operating light by the primary investigator (SD) and approved by the senior researcher (SGS). The time required for each tooth is noted in both groups.

The final measurement of surface roughness (T2)

**Figure 3:**
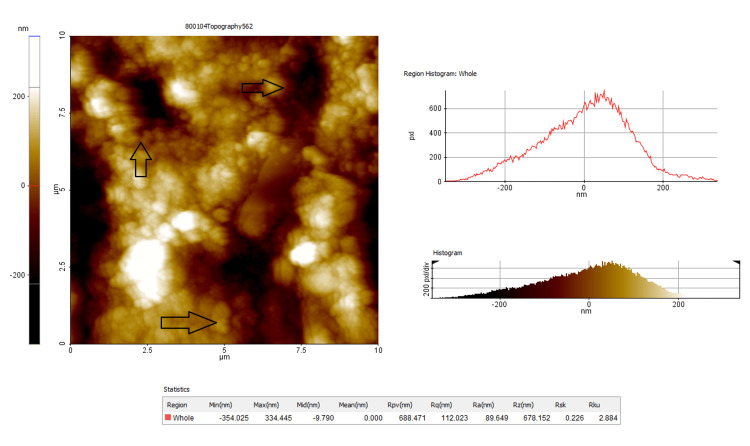
Topographic image and measurement of the enamel surface roughness characteristics of composite adhesive after bonding (T2). Arrow marks indicate increased surface roughness as well as surface roughness depth compared to RMGIC depth RMGIC: resin-modified glass ionomer cement.

**Figure 4:**
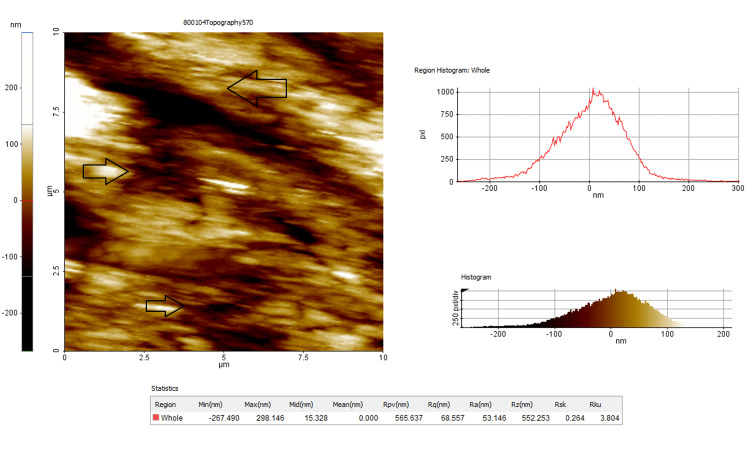
Topographic image and measurement of the enamel surface roughness characteristics of RMGIC adhesive group after bonding (T2). Arrow marks indicate less disturbance in the surface characters compared to the composite group. RMGIC: resin-modified glass ionomer cement.

At the end of the clean-up procedures (T2), the final roughness parameters, i.e. average roughness (Ra), root mean square roughness (Rq), and maximum roughness depth (Rmax), were measured through AFM (Figures 3, 4).

Data entry

The ARI scores were entered as frequency distribution. The time consumption for finishing in seconds and all the surface roughness parameters are measured as continuous data and entered as the mean for each of the test sample and the average mean and standard deviation for each of the group is calculated.

Statistical analysis

All the data assembled were entered into a Microsoft Excel sheet and then statistically analyzed with SPSS Statistics v.25 (IBM Corp, Armonk, NY). The ARI scores between the two groups were analyzed by the chi-square test. The mean difference in consumption time for the two groups is compared by Student’s independent t-test.

A two-way analysis of variance (ANOVA) was performed to determine if the type of adhesive and ARI scores had a significant effect on the time taken for the clean-up of the adhesive. The difference between the two groups was analyzed by Student’s independent t-test and intragroup comparative analysis was done with paired t-test. The probability (p) value for statistical significance was 0.05 or less for the difference between any two groups for all the analytical tests.

## Results

The distribution of the ARI scores of the two groups is shown in Tables 1, 2. The highest distribution for score 3 was noted for Group-I and score 5 for Group-II. The chi-square analysis shows that there is no statistical significance between the two groups regarding the frequency distribution of the different category scores (p > 0.05).

**Table 1:** Distribution of ARI scores for the two adhesive systems Modified ARI score: 1 = no adhesive remaining; 2 = less than 10% of adhesive remaining;  3 = 10-90% of adhesive remaining; 4 = over 90% of adhesive remaining; and 5 = all adhesive remaining on the tooth. ARI: adhesive remnant index; RMGIC: resin-modified glass ionomer cement. p^ns ^> 0.05: nonsignificant.

Groups	Sample size (n)	ARI scores					Chi-square value	p-Value
		1	2	3	4	5		
Group-I composite	20	1	3	8	3	5	7.267	0.122^ns^
Group-II RMGIC	20	1	1	2	6	10		
Column total		2	4	10	9	15		

**Table 2:** Tests of between-subjects effect two-way ANOVA. Dependent variable: time consumed in seconds. R-squared = 0.770 (adjusted R-squared = 9.720). *p < 0.05: significant; p^ns ^> 0.05: nonsignificant. Note: Post-hoc tests are not performed for adhesive because at least one group has fewer than two cases. ANOVA: analysis of variance; ARI: adhesive remnant index.

Source	Type III sum of squares	df	Mean square	F	p-Value
Corrected model	5281.176	7	754.454	15.33	<0.001*
Intercept	19312.032	1	19312.03	392.44	<0.001*
Adhesive	22.451	1	22.451	0.456	0.504^ns^
ARI	3945.61	3	1315.20	26.72	<0.001*
Adhesive * ARI	77.350	2	38.67 .	786	0.464^ns^
Error	1574.724	32	49.21		
Total	89848.000	40			
Corrected total	6855.900	39			

The two-way ANOVA shows that there was no statistically significant interaction (p = 0.464) effect between the type of adhesive and the ARI score. Simple main effects analysis revealed that adhesive (p = 0.504) had no statistically significant effect on time consumption whereas the ARI scores (p < 0.001) had a statistically significant effect on time consumption. 

The enamel surface roughness was captured as topographic images using AFM and the surface of the quality of the enamel was expressed as Ra, Rq, and Rz parameters, and the output was obtained in numerical values by the automated inbuilt mechanism of the system (Figures 3, 4). The mean values with a standard deviation of the enamel surface roughness before bonding and after debonding for composite adhesive and RMGIC adhesives were given. A statistically significant difference was noted in both groups when the intragroup comparison of enamel surface roughness before bonding (T1) and after debonding (T2) was analyzed (Tables 3, 4). 

**Table 3:** Mean enamel surface roughness and standard deviation in light cure composite group (Group-I; n = 20) - paired t-test Ra: average roughness; Rq: root mean square roughness; Rmax: maximum roughness depth; nm: nanometer; SD: standard deviation. *p < 0.05: significant.

Parameters	n	Mean ± SD	Mean difference	t-Value	p-Value
Ra (nm)					
Pre-bonding (T1)	20	42.22 ± 1.10	56.53	174.147	<0.001*
Post-debonding (T2)	20	98.75 ± 0.96			
Rq (nm)					
Pre-bonding (T1)	20	54.79 ± 1.30	65.58	175.468	<0.001*
Post-debonding (T2)	20	120.38 ± 1.06			
Rmax (nm)					
Pre-bonding (T1)	20	423.48 ± 13.14	226.65	76.685	<0.001*
Post-debonding (T2)	20	650.14 ± 1.12			

**Table 4:** Mean enamel surface roughness and standard deviation in RMGIC group (Group-II; n = 20) - paired t-test Ra: average roughness; Rq: root mean square roughness; Rmax: maximum roughness depth; nm: nanometer; SD: standard deviation. *p < 0.05: significant.

Parameters	Frequency	Mean ± SD	Mean difference	t-Value	p-Value
Ra (nm)					
Pre-bonding (T1)	20	42.14 ± 1.08	20.618	65.462	<0.001*
Post-debonding (T2)	20	62.76 ± 1.32			
Rq (nm)					
Pre-bonding (T1)	20	54.79 ± 1.11	26.422	99.124	<0.001*
Post-debonding (T2)	20	81.22 ± 1.13			
Rmax (nm)					
Pre-bonding (T1)	20	421.57 ± 1.02	12.787	33.544	<0.001*
Post-debonding (T2)	20	434.36 ± 1.60			

A comparison of enamel surface roughness between the groups before bonding is given in Table 5, and the values of roughness after finishing are given in Table 6. The results show that there is no statistical difference between the groups at the T1 stage, indicating a uniform distribution of the sample. However, there is a statistical difference between the groups at T2 (p < 0.001).

**Table 5:** Comparison of enamel surface roughness between the groups before bonding (T1) - independent Student's t-test Ra: average roughness; Rq: root mean square roughness; Rmax: maximum roughness depth; nm: nanometer; SD: standard deviation. p^ns^ > 0.05: nonsignificant.

Parameters	Group n = 20	Mean ± SD	Mean difference	t-Value	p-Value
Ra (nm)	Group-I	42.25 ± 1.10	0.081	0.236	0.815^ns^
	Group-II	42.14 ± 1.08			
Rq (nm)	Group-I	54.79 ± 1.30	0.001	0.003	0.998^ns^
	Group-II	54.79 ± 1.11			
Rmax (nm)	Group-I	423.48 ± 13.14	1.913	0.649	0.520^ns^
	Group-II	421.57 ± 1.02			

**Table 6:** Comparison of enamel surface roughness between the groups after debonding and polishing (T2) - independent Student's t-test Ra: average roughness; Rq: root mean square roughness; Rmax: maximum roughness depth; nm: nanometer; SD: standard deviation. *p < 0.05: significant.

Parameters	Group n = 20	Mean ± SD	Mean difference	t-Value	p-Value
Ra (nm)	Group-I	98.75 ± 0.96	35.99	98.391	<0.001*
	Group-II	62.76 ± 1.32			
Rq (nm)	Group-I	120.38 ± 1.06	39.15	112.490	<0.001*
	Group-II	81.22 ± 1.13			
Rmax (nm)	Group-I	650.14 ± 1.12	215.77	492.381	<0.001*
	Group-II	434.36 ± 1.60			

## Discussion

The surface of enamel is dented after orthodontic treatment, and it is inevitable regardless of the methods used; after bracket debonding and removal of adhesive on the enamel surface, scarring of the tooth is caused [16]. Plaque buildup due to the presence of resin remnant leads to the formation of decalcified areas and carious lesions leading to the unaesthetic appearance of the enamel as well as periodontal complications [17].

The development of dental materials, mainly resin composite as well as adhesive systems, has led to better enamel and resin adhesion, decreasing the bracket bonding failure rate for those undergoing orthodontic treatment [6]. The most efficient and safe debonding method is the one that removes maximum adhesive with reversible minimum damage to the enamel and leaves a finely polished enamel surface [7]. 

The most important factors involved in debonding are the type of bracket and adhesive used, the instruments used for bracket removal, and the armamentarium for resin removal. After debonding, the adhesive on the enamel surface can be removed in different ways including hand instruments (pliers and scalers), different types of burs, Sof-Lex discs, ultrasonic devices, and lasers. Enamel returns to its original condition as near as possible after debonding of orthodontic attachments if a specific method is followed to remove the remaining adhesive on the enamel surface [18]. Considering the results from the previous studies, it is for this reason we have selected a 12-fluted TC bur as a primary means of removal of both types of adhesives [2,3,8,18].

To overcome the problem of white spot lesions, RMGIC was introduced and it has been shown to release fluorides at much sustained and at higher levels than fluoride-releasing composites while maintaining adequate bond strength [19]. RMGIC is essentially a combination of composite resins and GIC. RMGICs have a diffusion-based adhesion between the cement and the tooth surface and continually release fluoride. They are autoset by the acid-base reaction of glass ionomer cement [20].

The shear bond strength, bracket failure, and bond strengths of stainless steel orthodontic brackets bonded with RMGIC were close to that of composite-based adhesives and were within the estimated range for successful clinical bonding [7,20-24].

Though the bond strength of the RMGIC adhesive is less than the composite resin, the added advantage of glass ionomer such as fluoride release overweighs the disadvantages. Further, it can be used on conditioned and unconditioned enamel surfaces [7,23,24]. It can be assumed from the above studies that the bond strength of the GIC cement may be at least equal or superior to that of composite resin for the placement of orthodontic brackets because it is less harmful to dental enamel. It possesses many favorable characteristics, including low solubility, excellent hardness, fluoride release, and good adhesive properties. Despite the many advantages of RMGICs, only a few studies were conducted on this adhesive for the evaluation of surface finish. So, this study was taken up to compare the effect of debonding and finishing procedures with two different types of adhesive systems - acid etching resin light cure-based adhesive and non-etching RMGIC.

ARI scores

The ARI values provide the mode of bracket failure during debonding. The modified version of the ARI index given by Bishara [15] was used to evaluate the bond failure according to the given scores: 1 = no adhesive remaining on enamel; 2 = less than 10% of adhesive remaining on enamel; 3 = 10-90% of adhesive remaining on enamel; 4 = over 90% of adhesive remaining on enamel; and 5 = all adhesive remaining on the tooth. 

The lower rank order indicates that the bond failure is at the enamel-adhesive surface interface and the higher score indicates that the bond failure is at the adhesive bracket interface. The bracket/adhesive interface can be considered the most favorable failure site for safe debonding, leaving most of the adhesive on the enamel surface, which is seen as scores 4 and 5. This interface can be considered safe since there is less chance of enamel fracture [25]. No significant difference was found between the groups when statistically analyzed for the type of bond failure during debonding. A lower score indicates less amount of enamel to be removed and hence less chance of damage to the enamel. However, there is a chance of enamel surface damage as bond failure occurs at the adhesive-enamel interface. However, there are different contradictory versions regarding the reports of whether low ARI scores are desirable or not [25]. One of the interesting findings of the present study was that despite the higher scores of ARI, a relatively smooth finish was obtained in Group-I compared to Group-II. 

Time duration of finishing

The average time required for the removal of adhesive in Group-I is 42 s, and in Group-II it is slightly higher at 49 s without any statistical and clinical significance. One of the recent studies concluded that the average time duration of composite removal with a TC bur was 33-34 s with or without loupes [26].

A two-way ANOVA was performed to determine if the type of adhesive and ARI scores had a significant effect on the time consumed for the clean-up of adhesive. There was no statistically significant interaction (p = 0.464) effect between the type of adhesive and the ARI score. Simple main effects analysis revealed that the adhesive (p = 0.504) had no statistically significant effect on time consumption whereas the ARI scores (p < 0.001) had a statistically significant effect on time consumption. Post hoc tests are not performed for adhesive because at least one group has fewer than two cases. Seventy-two percent of the variance in time consumption (adjusted R-squared = 0.720) is attributable to the variables and in particular to the ARI scores. 

Surface analysis

The surface analysis of enamel objectively assessed by an AFM is an excellent tool to study the morphology and texture of different surfaces [27]. This technique allows meticulous observations and evaluations of the textural and morphological characteristics of the enamel at nanometric resolution.

In the present study, the enamel surface roughness is evaluated using AFM. The AFM is essentially a scanning probe microscope that collects information on detected surfaces of enamel. The parameters that quantified the surface characteristics of the enamel are 1. Ra: Average roughness value is the arithmetic mean of the height of peaks and depth of valleys from a mean line; 2. Rq: Root mean square roughness is the height distribution relative to the mean line; and 3. Rmax: Maximum roughness depth value that represents isolated profile features.

The average roughness Ra values of the pretreatment enamel are around 42.12 nm at T1 and increased 2.33 times in Group-I and 1.48 times after debonding and finishing procedures. The values denote a rougher surface finish obtained with the composite material compared to RMGIC. The mean difference of change in Ra values is 56.53 nm in Group-I whereas it is only 20.61 nm in Group-II and this difference is statistically significant (p < 0.001).

Likewise, there is no significant difference in Rq and Rmax values between the two groups at T1 indicating a uniform distribution of the sample by randomization. The mean difference at T1 and T2 for composite adhesive is Rq (65.58 nm) and Rmax (226.65 nm), which is statistically significant (p < 0.001), compared to the GIC group with a mean difference of Rq (26.422 nm) and Rmax (12.787 nm). One of the reasons that may be attributed is the elimination acid etching step with RMGIC that resulted in less depth and surface area changes of enamel in this group. This indicates that the choice of RMGIC over composite is definitely a factor that increases the roughness of the enamel surface. This also indicates the application of secondary polishing and finishing techniques that should be considered to restore the enamel that was originally present at the beginning of the treatment.

The AFM method used in the present study has the advantage of analyzing both the qualitative and quantitative surface changes as compared to the SEM analyses [28]. The quality changes are visible with the composite resin showing a haphazard appearance and rougher surface compared to RMGIC. 

The Ra, Rq, and Rmax values obtained in the present study are in concordance with one of the earlier AFM studies of Sevinc [10] on composite bonding adhesive when finished with a TC bur. Their study reflected a pre-bond enamel (55.97 nm, 70.72 nm, and 394.38 nm) compared to post-debond and finish values (29.91 nm, 38.78 nm, 297.23 nm) of Ra, Rq, and Rmax, respectively. The number of flutes on the bur was not mentioned in their study. Another AFM study by Mohebi [11] showed that the estimated marginal change in the mean surface values (Ra) is about 20-40 nm when a 12-fluted TC was used and there was no statistically significant difference with and without loupes. However, there are no comparative studies available in the literature to make a direct comparison of the results obtained by the AFM method when RMGIC is used as one of the variables.

The results of the present study are not in tune with the recent clinical study which concluded that the choice of composite or RMGIC materials was not a factor that influenced the roughness of the enamel surface [26]. The central incisor teeth were used in their study as a test sample and the polishing variable was aluminum disks.

In this study, the null hypothesis was rejected as there is a significant difference in the surface properties after finishing between the groups tested as well as between pre-bond and post-debond times within each group.

Limitations

Basically, this is an in vitro study where the results have to be applied clinically very carefully. The surface finish of the enamel depends on many factors such as the type of adhesive, bonding protocols, type of curing, debonding methods and pliers, and the type of finishing and polishing. This study was conducted with only the adhesives as variables and keeping or assuming other factors to be constant. In our study, only the tungsten bur was evaluated and there is a need to evaluate the other systems. The bond strengths are not directly assessed in this study. The duration for the removal of resin remnants by using TC was not standardized. The surface finish after clean-up procedures was done by subjective assessment.

## Conclusions

Debonding and clean-up procedures should restore the surface characteristics of enamel to that of the pre-bond level. The surface roughness of the enamel definitely increases with the bonding and debonding procedures irrespective of the type of adhesive. This study advises the use of secondary finishing and polishing tools after the removal of adhesive with TC burs. The RMGIC adhesive results in less alteration of the enamel surface compared to the conventional light cure composite. If other conditions permit and if indicated, the use of RMGIC as a substitute for composite for orthodontic bonding may be considered in some clinical situations. 
